# Impact of resistance exercise on patients with chronic kidney disease

**DOI:** 10.1186/s12882-024-03547-5

**Published:** 2024-03-26

**Authors:** Chong-Cheng Chen, Yue-Yang Huang, Hua-Zhang, Xia-Liu, Xue-Qin Li, Yan-Qiong Long, Zheng-Wen Chen, Tao Jin

**Affiliations:** 1 Department of Nephrology, West China Hospital, Sichuan University, No.37 Guo Xue Xiang, Chengdu, Sichuan Province 610041 P.R. China; 2 West China School Of Medicine, West China Hospital, Sichuan University, No.37 Guo Xue Xiang, Chengdu, Sichuan Province 610041 P.R. China

**Keywords:** Resistance exercise, Glomerular filtration rate, C-reactive protein, Chronic kidney disease

## Abstract

**Background:**

Chronic kidney disease (CKD) has become an increasingly important public health disease with a high incidence rate and mortality. Although several studies have explored the effectiveness of resistance exercise in improving the prognosis of CKD patients, the number of studies is still limited and the results are still controversial.

**Objectives:**

We conducted this meta-analysis of randomized controlled trials (RCT) studies to evaluate the effectiveness of resistance exercise on CKD patients.

**Methods:**

The PubMed, Embase, and Cochrane Library databases were searched from the inception date to October 2023. The meta-analysis was conducted to evaluate 12 main indicators, including glomerular filtration rate (GFR)(ml/(min•1.73m2)), C-reactive protein (CRP) (mg/L), serum creatinine (mg/dL), hemoglobin (g/dL), Glycosylated Hemoglobin, Type A1C (HBA1c) (%), high Density Lipoprotein (HDL) (mg/dL), low Density Lipoprotein (LDL) (mg/dL), 6-min walk(m), body mass index (BMI) (kg/m^2^), fat-free mass (kg), fat mass (kg), grip strength (kgf).

**Results:**

Sixteen RCT studies were included in this meta-analysis from 875 records. GFR exhibited no significant change in CKD patients treated with resistance exercise (WMD 1.82; 95%CI -0.59 to 4.23; *P* = 0.139). However, 6-min walk (WMD 89.93; 95%CI 50.12 to 129.74; *P* = 0.000), fat-free mass (WMD 6.53; 95%CI 1.14 to 11.93; *P* = 0.018) and grip strength (WMD 3.97; 95%CI 1.89 to 6.05; *P* = 0.000) were significantly improved with resistance exercise. The level of CRP (WMD − 2.46; 95%CI -4.21 to -0.72; *P* = 0.006) and HBA1c (WMD − 0.46; 95%CI -0.63 to -0.29; *P* = 0.000) dropped significantly after resistance exercise treatment.

**Conclusions:**

Resistance exercise can improve physical function, metabolic condition, inflammatory response and cardiopulmonary function in CKD patients, specifically reflected in the increase of indicators fat-free mass, grip strength, 6-min walk, as well as the decrease of indicators HBA1c and CRP.

## Introduction

Chronic kidney disease (CKD) has become an increasingly important public health priority [1, 2]. Since 1990, the global all-age prevalence of CKD has increased by 29.3% [3]. Moreover, it is reported that 1.2 million people died from CKD globally in 2017 [3]. Chronic kidney disease is a progressive disease, which cannot be cured. It has a high incidence rate and mortality, and is common in the general adult population, especially in patients with diabetes and hypertension [4].

Long term chronic kidney disease can lead to many complications. Its main complication is cardiovascular disease (CVD), and it was reported that CKD is a strong independent risk factor of poor cardiovascular outcome [5–7]. It was reported that among patients aged 65 and above in the United States with CKD, the prevalence of CVD is 64.5%, while among patients without CKD, this proportion was only 32.4% [8]. Most patients with CKD will die due to cardiovascular disease before the CKD reaches its final stage [9]. In addition, CKD can also lead to complications such as metabolic syndrome and sarcopenia [10]. This process involves some complex molecular mechanisms, including reactive oxygen species (ROS) and NF-E2-related factor 2 (NRF2) [11]. According to previous studies, the incidence of muscle atrophy in CKD patients who started dialysis was 30%. There was also a significant correlation between the reduction of lean mass and the severity of kidney disease and physical function [12].

The impact of resistance exercise on improving the prognosis of CKD patients has also received increasing attention. For CKD patients, resistance exercise may be an ideal choice for improving prognosis. On the one hand, resistance exercise is not necessarily a very intense exercise that can cause harm to the body, including various types and forms (such as resistance bands, dumbbells) [13, 14]. On the other hand, resistance exercise can improve metabolic parameters with less energy consumption [15]. Therefore, resistance exercise may be more feasible for CKD patients, especially those with poor cardiopulmonary function. Moreover, resistance exercise can effectively alleviate the complications of CKD, including but not limited to metabolic syndrome, sarcopenic obesity, and reduce related biomarkers [16, 17].

The current evidence for resistance exercise treatment in CKD is encouraging. However, its practical application in clinical settings is still relatively limited [18]. We conducted this meta-analysis of existing literature on resistance exercise for the treatment of CKD, aiming to explore the effectiveness of resistance exercise in improving the prognosis of CKD patients.

## Methods

### Search strategy

According to PRISMA (Preferred Reporting Items for Systematic Reviews and Meta-Analyses) and AMSTAR (Assessing the Methodological Quality of Systematic Reviews) guidelines, the authors predetermined the eligibility criteria for the meta-analysis. We searched the PubMed, Embase, and Cochrane Library databases from the inception date to October 2023 using the keywords “resistance exercise”, “chronic kidney disease”, and “CKD” to identify published studies comparing patients with and without resistance exercise. We searched only English-language studies, and links within the search results and references were also examined to find additional literature. Grey literature was also reviewed. The primary outcome was assessed by glomerular filtration rate (GFR)(ml/(min•1.73m^2^)). The secondary outcomes were assessed by C-reactive protein (CRP) (mg/L), serum creatinine (mg/dL), hemoglobin (g/dL), Glycosylated Hemoglobin, Type A1C (HBA1c) (%), high Density Lipoprotein (HDL) (mg/dL), low Density Lipoprotein (LDL) (mg/dL), 6-min walk(m), body mass index (BMI) (kg/m^2^), fat-free mass (kg), fat mass (kg), grip strength (kgf).

### Eligibility criteria

In this study, two investigators independently reviewed all the literature and browsed the titles and abstracts of all papers. All the selected articles had to meet the following criteria: (1) Patients diagnosed with CKD; (2) Compared studies with and without resistance exercise; (3) studies that had sufficient data for statistical analysis. (4) The studies should be randomized controlled studies.

Articles including any of the following were excluded: (1) noncomparative studies such as meta-analyses, reviews, and case reports; (2) studies that were not focused on CKD patients underwent resistance exercise; (3) patients diagnosed with cancer, inflammatory disease, autoimmune disease; (4) duplicate studies.

### Data extraction and quality assessment

A thorough and independent review of the publications was conducted by two reviewers. Data extracted included the following items: author, year, study design, stage, age, number of female and male, dialysis duration, pathogen, GFR, CRP (mg/L), serum creatinine (mg/dL), hemoglobin (g/dL), HBA1c (%), HDL (mg/dL), LDL (mg/dL), 6-min walk(m), body mass index (BMI) (kg/m^2^), fat-free mass (kg), fat mass (kg), grip strength (kgf). The tool published by the Cochrane Collaboration in the Cochrane Handbook (version 5.3) was used to evaluate the risk of bias of one RCT and contained seven items: random sequence generation, blinding of participants and personnel, allocation concealment, blinding of outcome assessors, selective reporting, incomplete outcome data and other biases. An independent risk of bias assessment was carried out by two reviewers, and a third reviewer arbitrated unresolved differences. Potential biases were evaluated by 2 independent authors and were classified into three categories: “high risk”, “low risk”, and “unclear risk” according to the Cochrane bias risk assessment tool.

### Statistical analysis

Continuous variables were assessed using the weighted mean difference (WMD) and heterogeneity was assessed using the Q test with the corresponding *P* value and I^2^ test. It indicated that there was no heterogeneity if I^2^ was less than 25%; if 25% < I2 ≤ 50%, it implied moderate heterogeneity; if 50% < I2 ≤ 75%, it indicated substantial heterogeneity. If I2 ≥ 75%, it indicated considerable heterogeneity. An analysis of pooled data was conducted with a random-effects model if *P* > 0.05; otherwise, a fixed-effects model was applied. A funnel plot was used to assess publication bias. Egger’s test and Begg’s test were applied to determine publication bias. A sensitivity analysis was performed using the removal method. When studies provided only medians, we calculated the means, standard deviations and ranges. A statistically significant level of *P* < 0.05 was considered when analyzing the data using STATA version 15.0 software.

## Results

### Studies selected and characteristics

**Fig. 1:**
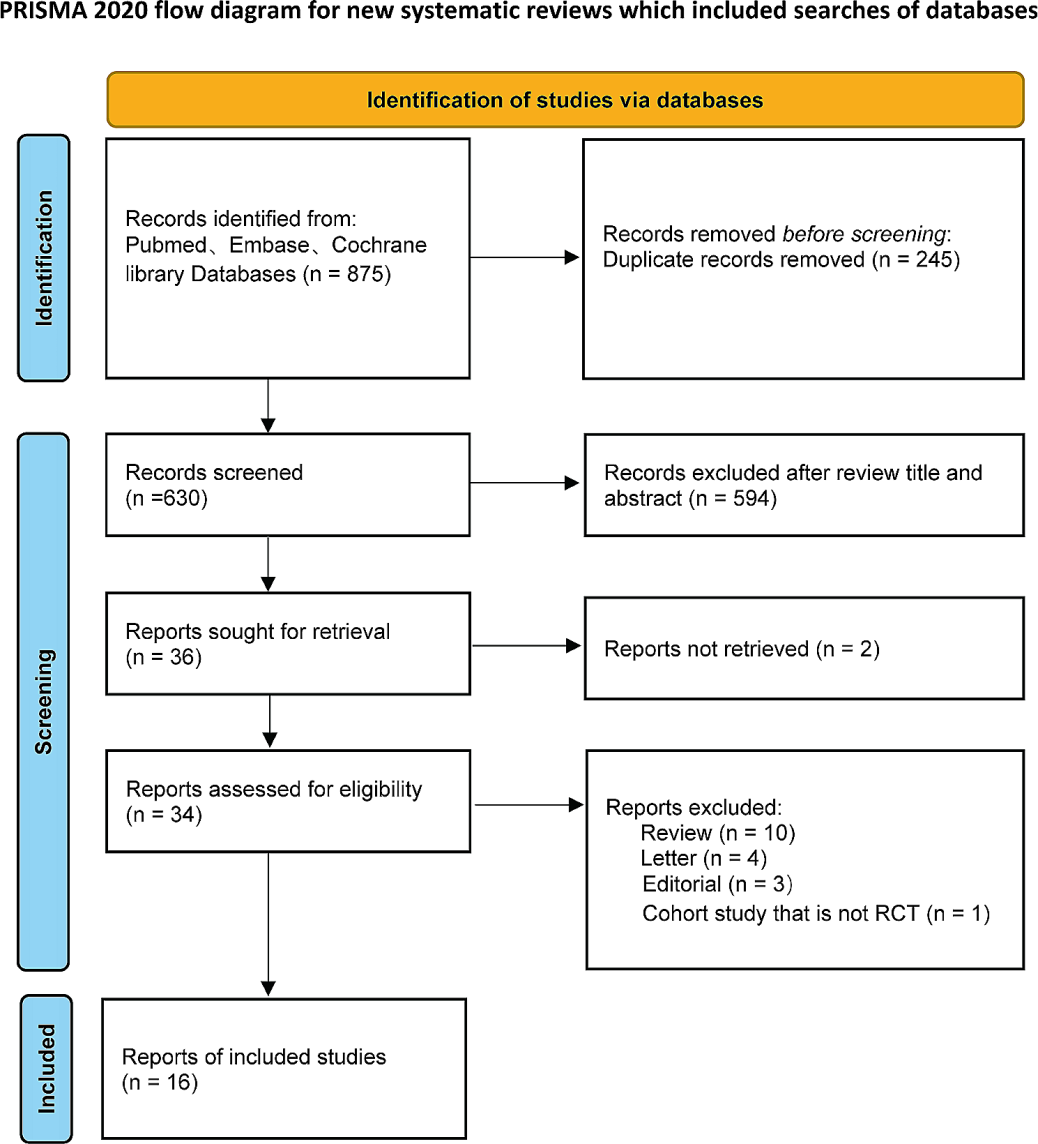
Flow chart of study screening and selection

A total of 875 potential articles published from the inception date to October 2023 were found. A total of 630 records remained after duplicates were removed. Thirty-six records were selected for further retrieval and evaluation after reviewing title and abstract. Two records were not retrieved. In addition, 10 reviews, 4 letters, 1 nonrandomized controlled trial study and 3 editorials were excluded during the assessment for eligibility. Eventually, 16 randomized controlled trial (RCT) studies met the criterion for our study and were included in the meta-analysis [19–34]. A corresponding flowchart is shown in Fig. 1.

A total of 787 CKD patients were included in this meta-analysis, of which the clearly reported causes were diabetes (*n* = 128), renovascular (*n* = 27), glomerulonephritis (*n* = 57), Polycystic kidney disease (*n* = 8). Seven articles clearly indicated the CKD stage of included patients. Fourteen studies described the gender ratio of the resistance exercise group and the control group. In general, the baseline features of the included studies are illustrated in Table 1.

**Table 1:** Basic characteristics of included studies

Author	Year	Stage	N	Age(I)	F/M(I)	BMI(I)	Age(C)	F/M(C)	BMI(C)	Duration	Diabetes	Renovascular	Glomerulonephritis	Polycystic kidney disease	other
Hugo	2021	2	60	58 ± 9	18/12	NA	57 ± 6	11/19	NA	6 months	NA	NA	NA	NA	NA
Erin	2015	3,4	72	60.2 ± 9.7	12/24	NA	62.0 ± 8.4	15/21	NA	12 months	20	11	21	3	17
Thaís	2022	2	31	57.94 ± 2.74	NA	26.64 ± 3.47	58.07 ± 5.22	NA	26.84 ± 1.95	22 weeks	NA	NA	NA	NA	NA
Sandra	2016	NA	13	71.5(58.5–87.2)	3/3	28.5(21.1–35.8)	76.0(59.0–83.0)	3/4	28.4(20.8–35.2)	8 weeks	NA	NA	NA	NA	NA
Abreu	2017	NA	44	45.7 ± 15.2	14/11	23.9 ± 4.7	42.5 ± 13.5	12/7	24.4 ± 4.8	6 months	NA	NA	NA	NA	NA
Barcellos	2018	2,3,4	150	65.0(1.2)	49/27	29.7(0.7)	65.1(1.3)	46/28	30.1(0.6)	16 weeks	NA	NA	NA	NA	NA
Carmen	2004	NA	26	65 ± 9	6/8	29.3 ± 6.6	64 ± 12	3/9	26.8 ± 2.7	12 weeks	NA	NA	NA	NA	NA
Kiyotaka	2021	4	46	72(69–79)	6/17	24.7 ± 4.6	76(69–78)	7/16	23.0 ± 4.3	6 months	14	12	NA	NA	NA
David J	2016	2,4	36	65.4 ± 8.7	0/18	36.2 ± 4.8	66.6 ± 7.5	0/18	37.4 ± 4.2	12 weeks	36	NA	NA	NA	NA
Samuel	2015	NA	46	58.0(8.0)	9/16	34.9(8.0)	57.1(9.0)	7/14	36.5(8.9)	14 weeks	2	NA	NA	NA	44
Jie	2011	NA	32	46.5 ± 12.1	6/9	27.5 ± 6.3	40.2 ± 13.5	5/12	29.1 ± 6.4	6 months	6	NA	NA	NA	26
Bobby	2007	NA	49	60.0 ± 15.3	7/17	74.9 ± 19.5	65.0 ± 12.9	8/17	76.5 ± 17.4	12 weeks	9	4	12	4	20
Ma	2016	NA	61	28.5(23-46.5)	16/14	21.8 ± 3.1	29(19–38)	12/19	21.1 ± 2.7	12 weeks	2	NA	NA	NA	NA
Song	2012	NA	40	52.1 ± 12.4	12/8	NA	54.6 ± 10.1	8/12	NA	12 weeks	22	NA	NA	NA	NA
Koji	2017	3,4	28	69.0 ± 6.8	NA	24.4 ± 3.5	67.8 ± 6.9	NA	23.0 ± 2.5	12 months	2	NA	8	1	17
Kumi	2021	NA	53	66.19 ± 13.05	6/20	22.52 ± 3.94	64.00 ± 12.95	6/21	23.30 ± 4.55	6 months	15	NA	16	NA	22

N: the total number of participants in the resistance exercise and control groups; Age(I): Age of the group of CKD patients with resistance exercise; F/M(I): The ratio of female to male patients of the group of CKD patients with resistance exercise; BMI(I): BMI of the group of CKD patients with resistance exercise; Age(C): Age of the group of CKD patients without resistance exercise; F/M(C): The ratio of female to male patients of the group of CKD patients without resistance exercise; BMI(C): BMI of the group of CKD patients without resistance exercise; Duration: Duration of resistance exercise intervention

### Quality assessment

**Fig. 2:**
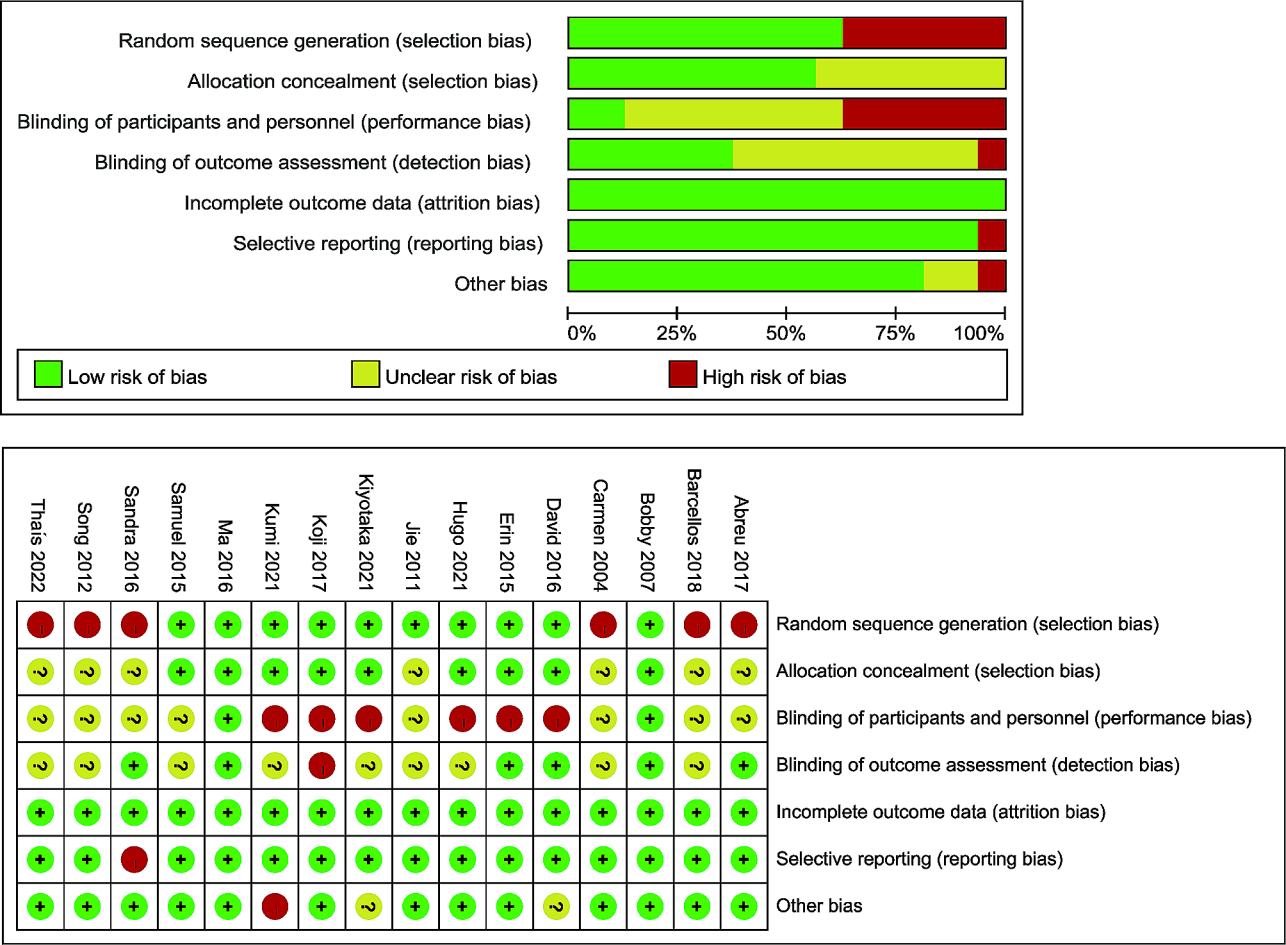
Quality assessment according to the Cochrane bias risk assessment tool

The evaluation of quality according to the Cochrane Bias Risk Assessment Tool is shown in Table 2; Fig. 2. Six studies were evaluated as high-risk on randomization. Six studies were identified as high-risk on blinding of participants and investigators. One study was considered high-risk on blinding of outcome assessment. In addition, 1 study exhibited high-risk on selective report of outcomes.

**Table 2:** The quality assessment of included studies of meta-analysis RCT quality assessment

Study	RANDOMISATION	ALLOCATION CONCEALMENT	BLINDING OF PARTICIPANTS AND INVESTIGATORS	BLINDING OF OUTCOME ASSESSMENT	INCOMPLETE OUTCOME DATA	SELECTIVE REPORT OF OUTCOMES	OTHER
Hugo	Low	Low	High	Unclear	Low	Low	Low
Erin	Low	Low	High	Low	Low	Low	Low
Thaís	High	Unclear	Unclear	Unclear	Low	Low	Low
Sandra	High	Unclear	Unclear	Low	Low	High	Low
Abreu	High	Unclear	Unclear	Low	Low	Low	Low
Barcellos	High	Unclear	Unclear	Unclear	Low	Low	Low
Carmen	High	Unclear	Unclear	Unclear	Low	Low	Low
Kiyotaka	Low	Low	High	Unclear	Low	Low	Unclear
David J	Low	Low	High	Low	Low	Low	Unclear
Samuel	Low	Low	Unclear	Unclear	Low	Low	Low
Jie	Low	Unclear	Unclear	Unclear	Low	Low	Low
Bobby	Low	Low	Low	Low	Low	Low	Low
Ma	Low	Low	Low	Low	Low	Low	Low
Song	High	Unclear	Unclear	Unclear	Low	Low	Low
Koji	Low	Low	High	High	Low	Low	Low
Kumi	Low	Low	High	Unclear	Low	Low	High

## Outcomes

### GFR

**Fig. 3:**
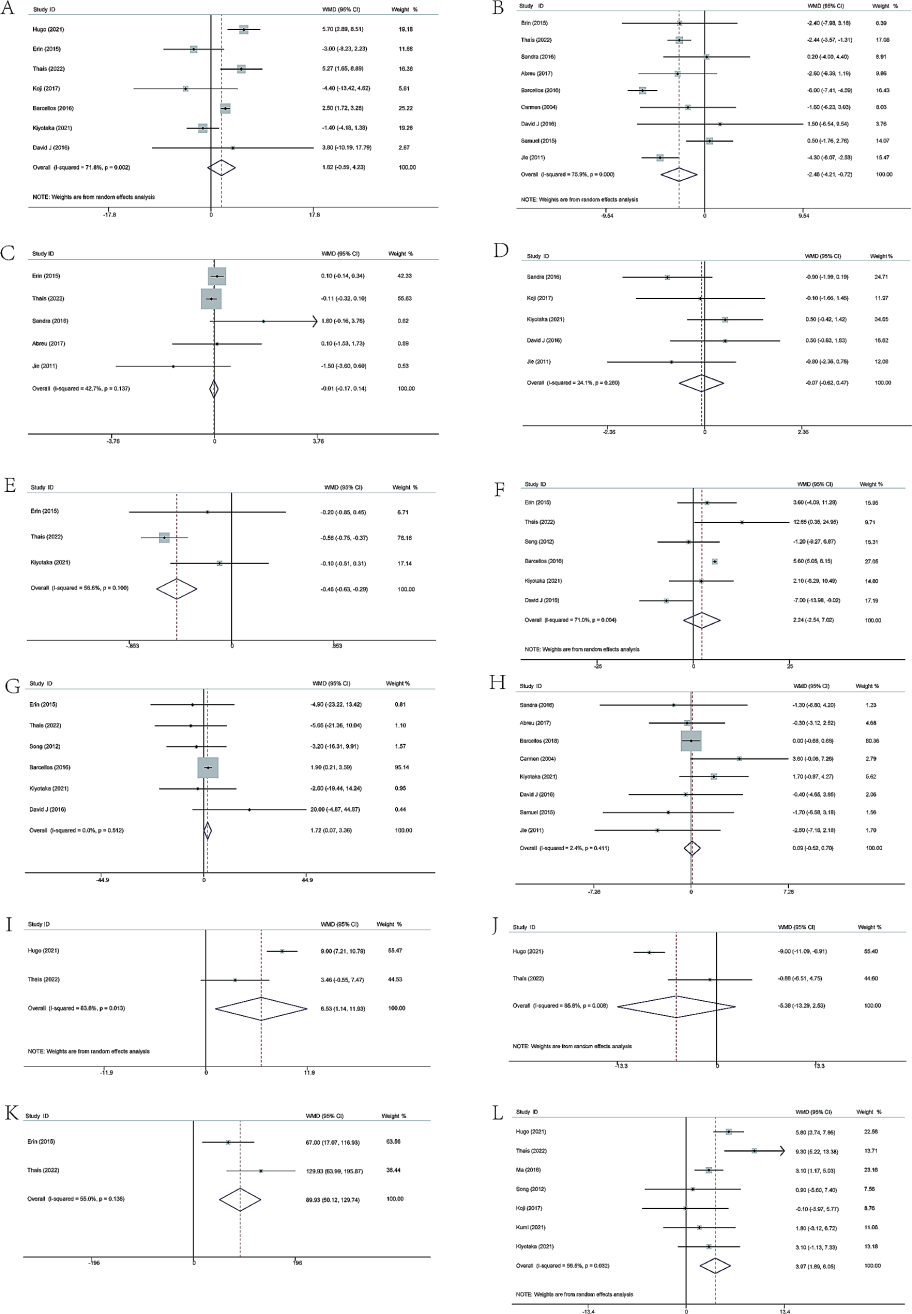
Forest plot of 12 indicators

The GFR was reported in 7 studies. The pooled outcome exhibited that there was no significant difference in GFR between CKD patients with and without resistance exercise (WMD 1.82; 95%CI -0.59 to 4.23; *P* = 0.139). The forest plot is illustrated in Fig. 3A.

### CRP

The CRP was examined in 9 records. The pooled effect revealed that the level of CRP in CKD patients with resistance exercise was significantly lower than that without resistance exercise (WMD − 2.46; 95%CI -4.21 to -0.72; *P* = 0.006). The forest plot is shown in Fig. 3B.

### Serum creatinine

The serum creatinine was detected in 5 trials. The level of serum creatinine in CKD patients with resistance exercise did not change significantly compared to that without resistance exercise (WMD − 0.01; 95%CI -0.17 to 0.14; *P* = 0.849). The corresponding forest plot could be seen in Fig. 3C.

### Hemoglobin

Five studies described the flow of the level of hemoglobin. No significant difference in the level of hemoglobin was found between CKD patients with and without resistance exercise (WMD − 0.07; 95%CI -0.62 to 0.47; *P* = 0.787). The forest plot is exhibited in Fig. 3D.

### HBA1c

HBA1c was mentioned in 3 studies. The pooled analysis suggested that the level of HBA1c in CKD patients met significant decrease when treated with resistance exercise (WMD − 0.46; 95%CI -0.63 to -0.29; *P* = 0.000). The forest plot is listed in Fig. 3E.

### HDL and LDL

HDL was presented in 6 studies. The level of HDL did not exhibit significant change when CKD patients were treated with resistance exercise (WMD 2.24; 95%CI -2.54 to 7.02; *P* = 0.359). In contrast, 6 studies reported the level of LDL. The pooled analysis illustrated the level of LDL with resistance exercise was significantly higher than that without resistance exercise in CKD patients (WMD 1.72; 95%CI 0.07 to 3.36; *P* = 0.512). The forest plot is shown in Fig. 3F and G.

### BMI, fat-free mass and fat mass

BMI was examined in 8 studies. The pooled outcome indicated that resistance exercise did not significantly reduce BMI in CKD patients (WMD 0.09; 95%CI -0.52 to 0.70; *P* = 0.776). The forest plot is shown in Fig. 3H.Fat-free mass was reported in 2 studies. The pooled analysis showed that the level of fat-free mass with resistance exercise was higher than that without resistance exercise (WMD 6.53; 95%CI 1.14 to 11.93; *P* = 0.018) (shown in Fig. 3I). 2 studies described the level of fat mass. The pooled effect showed no significant changes in fat mass when CKD patients were treated with resistance exercise (WMD − 5.38; 95%CI -13.29 to 2.53; *P* = 0.183) (shown in Fig. 3J).

### 6-min walk

Two studies presented the data of 6-min walk. The pooled analysis revealed that CKD patients with resistance exercise had a longer 6-min walk compared to the control group and the difference was statistically significant (WMD 89.93; 95%CI 50.12 to 129.74; *P* = 0.000). The forest plot is presented in Fig. 3K.

### Grip strength

Grip strength was evaluated in 7 studies. The grip strength of CKD patients in the resistance exercise group was significantly better than that of the control group according to the pooled outcome (WMD 3.97; 95%CI 1.89 to 6.05; *P* = 0.000). The forest plot is shown in Fig. 3L.

The pooled WMDs of all indicators included in this study are summarized in Table 3.

**Table 3:** The outcomes of pooled WMDs of meta-analysis

Overall	N	n (I/C)	WMD	95%CI	P (H)	I^2^	*P*
**GFR (ml/(min•1.73m** ^**2**^ **))**	7	191/185	1.82	-0.59 ~ 4.23	0.002	71.8%	0.139
**CRP (mg/L)**	9	202/187	-2.46	-4.21 ~ -0.72	0.000	75.9%	0.006
**Serum creatinine (mg/dL)**	5	93/89	-0.01	-0.17 ~ 0.14	0.137	42.7%	0.849
**Hemoglobin (g/dL)**	5	67/74	-0.07	-0.62 ~ 0.47	0.260	24.1%	0.787
**HBA1c (%)**	3	75/74	-0.46	-0.63 ~ -0.29	0.100	56.6%	0.000
**HDL (mg/dL)**	6	167/161	2.24	-2.54 ~ 7.02	0.004	71.0%	0.359
**LDL (mg/dL)**	6	165/159	1.72	0.07 ~ 3.36	0.512	0.0%	0.040
**6-min walk (m)**	2	52/51	89.93	50.12 ~ 129.74	0.136	55.0%	0.000
**BMI (kg/m** ^**2**^ **)**	8	175/162	0.09	-0.52 ~ 0.70	0.411	2.4%	0.776
**Fat-free mass (kg)**	2	46/45	6.53	1.14 ~ 11.93	0.013	83.6%	0.018
**Fat mass (kg)**	2	46/45	-5.38	-13.29 ~ 2.53	0.008	85.8%	0.183
**Grip strength (kgf)**	7	159/160	3.97	1.89 ~ 6.05	0.032	56.5%	0.000

N: number of included studies; n (I/C): number of patients of intervention (resistance exercise) group and control group (I for intervention group; C for control group); WMD: weighted mean difference; P (H): *p* value for heterogeneity; P: *p* value for pooled WMD effect; GFR: glomerular filtration rate; CRP: C-reactive protein; HBA1c: glycosylated hemoglobin, type A1c; HDL: high-density lipoprotein; LDL: low-density lipoprotein; BMI: body mass index

### Sensitivity analysis and publication bias

**Fig. 4:**
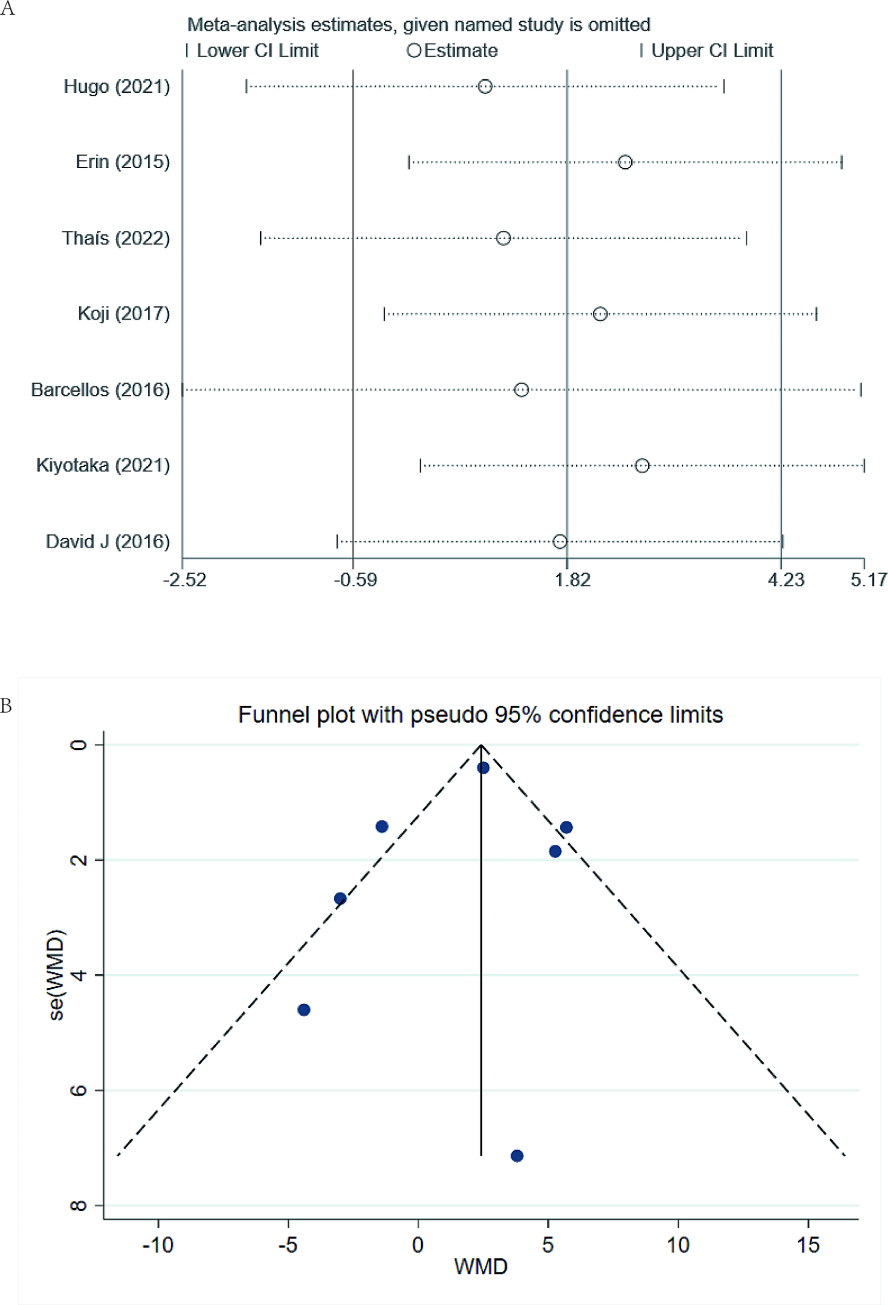
Sensitivity analysis and publication bias assessment

Sensitivity analysis revealed that the pooled WMDs were not materially influenced by any single study in all outcomes, indicating that the outcomes were statistically robust (shown in Fig. 4A).

Publication bias was evaluated by using a funnel plot visually (shown in Fig. 4B), which indicated no significant publication bias. Statistically, Egger’s test also suggested no significant publication bias though outcomes with less than 10 included studies (*P* = 0.625).

## Discussion

This meta-analysis demonstrated the comparisons of 12 outcomes between CKD patients with resistance exercise treatment and those without resistance exercise. The primary outcome, the level of GFR, was not improved significantly after resistance exercise in CKD patients (WMD 1.82; 95%CI -0.59 to 4.23; *P* = 0.139). The secondary outcomes, including the level of serum creatinine, hemoglobin, HDL, BMI and fat mass, did not change significantly when CKD patients were treated with resistance exercise. Moreover, the level of LDL, 6-min walk, fat-free mass and grip strength increased significantly with resistance exercise, while the level of CRP and HBA1c dropped significantly with resistance exercise.

This meta-analysis did not demonstrate the beneficial effects of resistance exercise on renal function, which was consistent with the results of some other RCT studies [28, 33]. The changes in GFR and serum creatinine of patients receiving resistance exercise treatment were not significant compared to that of the control group. The impact of resistance exercise on GFR in CKD patients is still controversial. The research data of Corrêa, H. L. et al. [23] confirms that resistance exercise could alleviate the decrease in GFR in CKD patients, while the research data of Erin et al. [28] could not support this outcome. A study on resistance exercise for the elderly showed that resistance exercise programs can improve GFR [35]. However, the study by Amorim and Machado et al. showed that at 24, 48, and 72 h after resistance exercise, serum creatine kinase (CK) activity was elevated while eGFR dropped, showing a negative correlation between the two [36, 37]. Only 7 studies included in the meta-analysis recorded the GFR indicator. RCT studies on the impact of resistance exercise on renal function are still limited and need further research.

However, it is encouraging to note that this meta-analysis found that resistance exercise can improve physical function, metabolic condition, inflammatory response and cardiopulmonary function in CKD patients. Resistance exercise could increase the 6-min walk, fat-free mass and grip strength of the CKD patients significantly according to our meta-analysis. The increase in distance of 6-min walk indicated an improvement in cardiopulmonary function in CKD patients after receiving resistance exercise. Half a SD (standard deviation) was reported to be the estimated MCiD (minimal clinically important difference) for chronic diseases [38]. Therefore, we used half a SD to calculate the estimated MCiD. The WMD of 6-min walk (89.93) was greater than the estimated MCiD (20.31), indicating that this difference is not only statistically significant but also clinically significant. Moreover, the increase in fat-free mass and grip strength suggested an enhancement in muscle mass and muscle strength, respectively. The estimated MCiD of fat-free mass and grip strength were 2.75 and 1.06, respectively, suggesting that the increase in fat-free mass and grip strength were both clinically significant. The above three outcomes suggested that resistance exercise could improve the physical function of CKD patients. Moreover, resistance exercise can significantly reduce the levels of CRP and HBA1c in CKD patients according to our meta-analysis. CRP is not only an inflammatory marker, but also a predictor and risk factor for cardiovascular disease [39]. CRP, whose MCiD was − 0.89, showed a significant drop (WMD − 2.46; 95%CI -4.21 to -0.72; *P* = 0.006) indicating a relief of the level of inflammation and the risk of cardiovascular complications in CKD patients after resistance exercise, which revealed resistance exercise can alleviate the inflammatory response of CKD and improve the prognosis of CKD patients. In addition, the decrease in HBA1c ratio (WMD − 0.46; 95%CI -0.63 to -0.29; *P* = 0.000), which exceeded its corresponding estimated MCiD (-0.09), indicated that CKD patients can improve their metabolic condition and alleviate the progression of CKD by resistance exercise in controlling blood sugar and other aspects. However, it is worth noting that CKD patients showed a significant elevation in LDL after resistance exercise, which is one of the risk factors for cardiovascular disease. We subsequently conducted a sensitivity analysis on the included studies that recorded LDL, and the result showed that excluding the study by Barcellos et al., the pooled outcome changed significantly, indicating that the study by Barcellos et al. influenced the stability of the pooled outcome [20]. We excluded the research data from Barcellos et al. and conducted a meta-analysis again. The pooled effect showed that there was no significant change in LDL levels in CKD patients after resistance exercise (WMD − 1.83; 95%CI -9.29 to 5.62; *P* = 0.630). Overall, this meta-analysis suggested that resistance exercise could improve the physical function and metabolic condition of CKD patients.

### Research and clinical implications

Resistance exercise has been reported to have beneficial effects on physical function, metabolic condition, inflammatory response and cardiopulmonary function in adults with CKD, which is consistent with our meta-analysis outcomes [17, 18, 40]. However, the mechanisms behind these outcomes have received little attention [40]. ROS and NRF2 might be key molecules in the progression of CKD [11, 19, 40]. The kidney is one of the organs with the highest oxygen consumption. The kidneys only account for 0.5% of total body weight, but they account for approximately 7% of the body’s total oxygen consumption [11]. Mitochondria is one of the important sources of endogenous ROS in the kidney. Mitochondria in kidney cells may leak some electrons during electron transport, leading to the production of superoxide. Usually, the generation and elimination of ROS reach a balance. But in CKD patients, this balance is disrupted, and excessive ROS ultimately leads to oxidative stress [10]. The oxidative stress leads to a decrease in the degradation of NRF2, which transfers from the cytoplasm to the nucleus, inducing the expression of glutathione synthase and HO1, which are crucial for protecting cells and organs from oxidative stress. However, in animal models of CKD, a decrease in NRF2 activity was observed in the kidneys [41]. The weakened antioxidant stress capacity of the kidneys ultimately leads to the progression of CKD. The progression of CKD leads to a decline in physical function, deterioration of metabolic condition [42], increased inflammatory response, and impaired cardiopulmonary function in CKD patients, while resistance exercise could alleviate these complications. For physical function, resistance exercise could decrease the expression of myostatin and elevate the expression of insulin-like growth factor-1 (IGF-1), which could improve the synthesis of protein in skeletal muscle and attenuate protein degradation [12, 40]. Moreover, resistance exercise is proved to increase the expression of peroxisome proliferator-activated receptor coactivator-γ-1α4 (PGC-1α4, an isoform of the transcriptional co-activator PGC-1α), which not only enhances muscle hypertrophy but also improves glycolysis [43]. For metabolic condition, resistance exercise is found to improve the bind between PGC-1α4 and a nuclear receptor named peroxisome proliferator-activated receptor β (PPARβ), enhancing anaerobic glycolysis, which could promote the glucose uptake and fat oxidation in skeletal muscle [43]. As a result, resistance exercise is beneficial for improving glucose and lipid metabolism. For inflammatory, NRF2 has been mentioned as a potential key molecule in the impact of resistance exercise on the progression of CKD. Resistance exercise could improve the expression of NRF2 thereby alleviate oxidative stress and inflammatory response [19]. For cardiopulmonary function, resistance exercise improves the muscular contractions, resting heart rate and blood pressure, resulting in shear stress-induced adaptations in nitric oxide metabolism. Therefore, resistance exercise might play a critical role in improving flow-mediated dilatation, enhancing cardiopulmonary function and reducing the risk of cardiovascular diseases [44]. In summary, resistance exercise improves the prognosis of CKD patients by affecting the expression and activity of multiple molecules, including ROS, NRF2, IGF-1 and PGC-1α4. Moreover, further research is needed to verify the changes in other indicators. Different stages and causes of CKD may affect the effectiveness of resistance exercise in improving CKD prognosis and alleviating CKD progression.

## Conclusion

This meta-analysis shows that resistance exercise can improve physical function, metabolic condition, inflammatory response and cardiopulmonary function in CKD patients, specifically reflected in the increase of indicators fat-free mass, grip strength, 6-min walk, as well as the decrease of indicators HBA1c and CRP.

## Data Availability

All data analyzed in this study are included in this published article.
